# Short report: relationship between restricted and repetitive behaviours in children with autism spectrum disorder and their parents

**DOI:** 10.1186/s13229-016-0091-y

**Published:** 2016-06-14

**Authors:** Mirko Uljarević, David W. Evans, Gail A. Alvares, Andrew J. O. Whitehouse

**Affiliations:** Olga Tennison Autism Research Centre, School of Psychology and Public Health, La Trobe University, Bundora, Victoria 3086 Australia; Department of Psychology, Bucknell University, Lewisburg, PA USA; Telethon Kids Institute, University of Western Australia, Perth, WA Australia; Cooperative Research Centre for Living with Autism (Autism CRC), Long Pocket, Brisbane, Queensland Australia

**Keywords:** Autism, Repetitive behaviours, Broader Autism Phenotype, Parents

## Abstract

**Background:**

Restricted and repetitive behaviours (RRBs) constitute a core symptom domain of autism spectrum disorder (ASD). However, the nature of RRBs in the context of the Broader Autism Phenotype (BAP) is not well understood. In particular, the relationship between RRBs in ASD probands and their parents remains largely unexplored. The current study explored the link between parental RRBs, measured via Interest in Patterns and Resistance to Changes subscales of the Autism Quotient and their children’s RRBs, measured via Autism Diagnostic Observation Schedule RRB standardized domain score.

**Findings:**

Having both parents within the top 20 % of their RRB scores was associated with an increase of RRB scores for their children; however, no parent-of-origin effects were identified. Although the trend was observed for both Interest in Patterns and Resistance to Changes subscale, it was only statistically significant for Interest in Patterns.

**Conclusions:**

This paper provides significant contribution to our understanding of association between RRBs in parents and their children with ASD. Future work should also address the BAP in distinct genetic subtypes (whole chromosome aneuploidies, single gene mutations, copy number variations) of neurodevelopmental and neuropsychiatric disorders that involve RRBs.

## Background

Autism spectrum disorder (ASD) is a behaviourally defined cluster of neurodevelopmental conditions considered to be amongst the most heritable complex neuropsychiatric disorders. Family and twin studies suggest that the recurrence rate of ASD between siblings is between 3 and 14 % [1] and that the concordance of ASD is 58 % for male and 60 % for female monozygotic and 21 % for male and 27 % for female dizygotic pairs [2]. Since the original descriptions by Kanner [3] and Asperger [4], it has been recognized that family members of affected individuals—in particular parents—in addition to having high recurrence of categorical ASD diagnosis, show traits and behaviours that are qualitatively similar to those seen in ASD, but less severe. This presence of ASD traits exhibited to a lesser degree is commonly referred to as the Broader Autism Phenotype (BAP) [5, 6].

Previous research has mainly concentrated on the socio-communicative aspects of the BAP. When compared to family members of control individuals, relatives of individuals with ASD, from high infant siblings to parents, tend to show higher levels of language, communication, social interaction, and cognition difficulties [7, 8]. Evidence of familiality and inter-generational transmission of these traits has also been reported [9–12].

Restricted and repetitive behaviours (RRBs) constitute a core symptom domain of ASD. However, while RRBs represent one of two core symptom clusters in the the 5th edition of the Diagnostic and Statistical Manual of Mental Disorders criteria for ASD (DSM-5) [13], the nature of RRB in the context of the BAP is not well understood. Research to date suggests elevated levels of RRBs in unaffected family members. However, most studies have used measures that assess personality traits and activities that are indicative of potential RRBs [14–17] rather than dedicated RRB measures [18, 19]. Although several studies have explored evidence for familiality of RRBs amongst affected siblings with ASD and high risk siblings without the disorder [20–24], the relationship between RRBs in ASD probands and their parents remains largely unexplored. The paucity of data on the associations between RRBs in ASD probands and their parents is a significant limitation in the scholarly literature. The only exception is a study by Smith et al. [25] who found that intense preoccupations (as measured via ADI-R) in ASD probands were associated with rigidity and aloofness in their fathers; however, as with studies reviewed above, Smith and colleagues have used parental personality traits as a proxy of RRBs rather than a dedicated measure.

The current study explored relationships between parental and proband RRBs. Based on evidence from studies of socio-communicative behaviours, we hypothesized that higher RRBs scores in parents would be associated with higher levels of RRBs in their children diagnosed with ASD, but not in higher levels of socio-communicative problems. We made no specific predictions in terms of the parent-of-origin effect.

## Methods

### Participants

One hundred and sixty-nine mother-father-child with ASD triads (mother, *M*_age_ = 39.73 years, SD_age_ = 6.38; father, *M*_age_ = 41.76 years, SD_age_ = 6.82; child, *M*_age_ = 8.03 years, SD_age_ = 3.91, range, 2.43–17.8, 138 males) participated as a part of the Western Australian Autism Biological Registry (WAABR) study. All children had a community diagnostic assessment conducted by a multidisciplinary team comprising a paediatrician, clinical psychologist, and speech pathologist, leading to a best-estimate clinical diagnosis of an ASD. At their research appointment for WAABR, the child’s diagnosis was verified using the Autism Diagnostic Observation Schedule-Generic (ADOS-G) [26].

### Procedures and measures

The study was approved by the Princess Margaret Hospital Human Research Ethics Committee, and all participants have provided informed consent. Parents were mailed the Autism Spectrum Quotient (AQ) [27] as a part of a series of questionnaires included in the WAABR study. Families then attended the Telethon Kids Institute in Perth, Western Australia, where the ADOS-G was administered by the same trained and research-reliable assessor.

The AQ is a self-report questionnaire designed to measure levels of autistic-like traits. In this study, we have used Interest in Patterns (IP; five items, example item: ‘I like to collect information about categories of things’; Cronbach’s *α* = .71) and Resistance to Change (RC; five items, ‘I prefer to do things the same way over and over again’; Cronbach’s *α* = .73) factors identified by Lau et al. [28] in a sample of Australian adults with and without ASD as measures of parental RRBs.

The ADOS-G is a clinician-administered observational assessment developed as a standardized classification of ASD. Forty-nine children were administered ADOS-G module 1, 62 Module 2, 55 Module 3, and 3 Module 4. We have used standardized ADOS-G scores for the social affect (SA) and RRBs domains following Hus et al. [29], as these scores can be used across individuals of different developmental levels.

## Results

RRB scores between probands whose mother or father had elevated RRB scores were first compared in order to explore potential parent-of-origin effects. Following the methodology used by Lyall et al. [12], elevated RRB scores in parents (IP and RC were considered separately) were defined as the top 20 % of the score distribution for mothers and fathers, respectively. There was no evidence of parent-of-origin effect when either IP *U* = 335.5, *z* = −.378, *p* = .705 or RC *U* = 463.5, *z* = −.007, *p* = .994 scores were used to stratify the sample into children whose only mother had scores in the top 20 %. Mann-Whitney *U* test revealed no significant difference in ADOS RRB scores between male and female probands *U* = 2133.5, *z* = −.023, *p* = .982. For these reasons, we partitioned ASD probands based on whether neither parent had elevated RRB scores, one parent (either mother or father) had elevated RRB scores, or both parents had had elevated RRB scores. As can be seen from the Table 1, when probands were grouped based on their parental IP scores, there was a significant difference on ADOS RRBs (*p* = .018).

**Table 1:** Means (and SDs) ADOS standardized domain scores as a function of parental RRB status

	Parent with elevated RRB traits						
	Neither (N; *N* = 96)	Either (E; *N* = 53)	Both (B; *N* = 18)	*χ* ^2^	*p*	*ω* ^2^	Post hoc (Mann-Whitney *U*)
AQ Interest in Patterns							
ADOS SA	7.52 (2.07)	7.28 (2.13)	7.83 (1.82)	.960	.62	0	NS
ADOS RRBs	3.98 (2.35)	4.42 (2.56)	5.67 (2.19)	8.08	.018	.03	B > N (*p* = .002)
AQ Resistance to Change							
ADOS SA	7.44 (2.21)	7.41 (1.95)	8 (1.56)	.94	.62	0	NS
ADOS RRBs	4.13 (2.33)	4.38 (2.52)	5 (2.8)	1.25	.53	0	NS

Note: elevated RRB traits are defined as the top 20 % of the score distribution

Although a similar trend was observed when RC parental scores were used to group ASD probands, there were no significant differences between groups in terms of ADOS RRB (*p* = 53). No significant differences in terms of ADOS SA scores were observed, either when parental IP (*p* = .62) or RC scores (*p* = .62) were used as a grouping variable.

## Discussion

The current study explored the link between parental RRBs, measured via Interest in Patterns and Resistance to Changes subscales of the AQ and their children’s RRBs, measured via ADOS RRB standardized domain score. Having both parents within the top 20th percentile of their RRB scores was associated with an increase of ADOS RRB scores for their children; however, no parent-of-origin effects were identified. Although the trend was observed for both IP and RC RRBs, it was only significant for IP but not for RC. This finding is broadly in line with findings by Smith et al. [25] who reported that increased IP but not RC in ASD probands were associated with parental personality traits indicative of RRBs. In line with the current understanding of the ASD traits as being fractionable at the genetic level [30], no such trend was observed for the social affect ADOS domain.

Our study had an advantage of using observational measure of RRBs in children rather than parental report, thus avoiding potential issues related to shared method variance and reporter bias. Another advantage was using measures that assess RRBs in both parents and their children, rather than personality, as has been the case in the only study to date that has explored the relationship between RRBs in probands with ASD and their parents [25]. However, both ADOS and AQ are limited in their sampling of RRBs. The AQ reflects only what can be considered ‘high level’ (i.e. compulsive behaviour) RRBs, whereas the ADOS-G provides only an overall score for RRBs. Furthermore, although the observational context that ADOS provides is conducive to noting motor stereotypes and some sensory interests, it is limited in its ability to assess more complex restricted patterns of interest and rigid types of behaviours/insistence on sameness. For assessing these behaviours, sampling across time and contexts is necessary. This limited sampling is a significant issue as, despite all RRBs being linked by underlying problems of rigidity, repetitiveness, invariance and inappropriateness, they are an extremely heterogeneous group ranging from simple motor tics to extremely complex patterns of routinized behaviours and interests. Therefore, it has been suggested that RRBs should be treated as multidimensional construct that encompasses several related but distinct behavioural categories [31, 32].

Instruments designed for purposes of screening and diagnosis of ASD are limited by concentrating only on behaviours considered to be specific to ASD despite RRBs cutting across a various wide range of neurodevelopmental, neuropsychiatric and neurological disorders (e.g. schizophrenia, obsessive-compulsive disorder, Parkinson disease, Sydenham chorea, Tourette syndrome, ASD; [32]) and RRBs also being present during typical development [33–35]. It has been argued (for example, Research Domain Criteria (RDoC); [36]) that rather than studying particular behaviours within categorically defined clinical diagnoses, it is necessary to study these behaviours across both typical development and disorders in order to be able to identify their genetic, neural underpinnings and mechanisms. In order to achieve this, RRB measures need to be comprehensive enough to gather information on a wide range of behaviours that are not exclusive to any particular disorder, be appropriate for use across the lifespan, and be sensitive enough to identify mild variants of symptom expression. Although reliable measures of RRBs such as the Repetitive Behaviour Qestionnaire-2 [35, 37] and Repetitive Behaviour Scale-Revised [Bodfish JW, Symons FW, Lewis MH. The Repetitive Behavior Scale. Western Carolina Center Research Reports (unpublished). 1999] are available, they do not meet these criteria. Development of quantitative trait assessment tools for RRBs is therefore a key for future research.

## Conclusion

This paper provides significant contribution to our understanding of association between RRBs in parents and their children with ASD. The data extend previous research by using dedicated RRB measures for both parents and children. Further research will need to explore the pattern of inter-generational transmission of different types of RRBs across both normative development and clinical condition using reliable, quantitative trait measures of RRBs. Future work should also address the BAP in distinct genetic subtypes (whole chromosome aneuploidies, single gene mutations, copy number variations) of neurodevelopmental and neuropsychiatric disorders that involve RRBs.

## Abbreviations

ADOS, Autism Diagnostic Observation Schedule; AQ, Autism Spectrum Quotient; ASD, autism spectrum disorder; BAP, Broader Autism Phenotype; IP, Interest in Patterns; RC, Resistance to Changes; RRBs, restricted and repetitive behaviours; WAABR, Western Australian Autism Biological Registry
